# Open IoT Ecosystem for Enhanced Interoperability in Smart Cities—Example of Métropole De Lyon

**DOI:** 10.3390/s17122849

**Published:** 2017-12-08

**Authors:** Jérémy Robert, Sylvain Kubler, Niklas Kolbe, Alessandro Cerioni, Emmanuel Gastaud, Kary Främling

**Affiliations:** 1 Interdisciplinary Center for Security, Reliability and Trust, University of Luxembourg, 29 Avenue J.F. Kennedy, Luxembourg L-1855, Luxembourg; niklas.kolbe@uni.lu; 2 Université de Lorraine, CRAN, UMR 7039, 2 Avenue de la forêt de Haye, Vandoeuvre-lès-Nancy CEDEX 54516, France; s.kubler@univ-lorraine.fr; 3 CNRS, CRAN, UMR 7039, France; 4 Direction de l’Innovation Numérique et Systèmes d’Information, Métropole de Lyon, 20 rue du Lac, CS 33569, 69505 Lyon CEDEX 3, France; egastaud@grandlyon.com (A.C.); acerioni@grandlyon.com (E.G.); 5 School of Science and Technology, Aalto University, P.O. Box 15500, Aalto 00076, Finland; kary.framling@aalto.fi

**Keywords:** smart city, Internet of Things, interoperability, semantic web, communication standards, API economy, business ecosystem, open innovation

## Abstract

The Internet of Things (IoT) has promised a future where everything gets connected. Unfortunately, building a single global ecosystem of Things that communicate with each other seamlessly is virtually impossible today. The reason is that the IoT is essentially a collection of isolated “Intranets of Things”, also referred to as “vertical silos”, which cannot easily and efficiently interact with each other. Smart cities are perhaps the most striking examples of this problem since they comprise a wide range of stakeholders and service providers who must work together, including urban planners, financial organisations, public and private service providers, telecommunication providers, industries, citizens, and so forth. Within this context, the contribution of this paper is threefold: (i) discuss business and technological implications as well as challenges of creating successful open innovation ecosystems, (ii) present the technological building blocks underlying an IoT ecosystem developed in the framework of the EU Horizon 2020 programme, (iii) present a smart city pilot (Heat Wave Mitigation in *Métropole de Lyon*) for which the proposed ecosystem significantly contributes to improving interoperability between a number of system components, and reducing regulatory barriers for joint service co-creation practices.

## 1. Introduction

Over the past decade, a flourishing number of concepts and economic shifts appeared such as cloud computing, Internet of Things (IoT), Semantic Web, or still the sharing economy [1,2,3]. This evolution opens up the way for new applications that leverage ubiquitous connectivity and analytics in a wide range of sectors (e.g., transportation, energy, manufacturing, healthcare, cities, etc.), as well as for disruptive innovation and business models that reduce costs for societies, foster a sustainable economic growth, and improve the quality of citizen-oriented services. Ideally, the IoT should provide means to create, when and as needed, ad hoc and loosely coupled information flows between any kinds of smart connected objects, databases and users. Nonetheless, while new smart connected objects hit the market every day, they mostly feed “vertical silos” that are closed to the rest of the IoT [4,5] (e.g., we still need to install ten different apps to interact with ten different devices). This ineluctably leads to the emergence of isolated and protected islands of information, which entails the development of competition in the IoT market [6]. As stated by Guinard and Trifa [7], the problems is that there is no *lingua franca* spoken by each and every object, there are literally hundreds! The worst part is that most of these IoT protocols and standards are not compatible with each other, which is one of the key reasons why the IoT has not (yet!) delivered on its promises. IoT integrator companies estimate that support for every new service API requires a few days to months of software development effort [8], not to mention the effort to maintain these APIs.

Several organisms and standardization fora understood this critical challenge and started to build up consortia and initiatives such as for instance, the *Web of Things* initiative at W3C that aims to create open ecosystems based upon open standards, including identification, discovery and interoperation of services across platforms [9]; the *Alliance for Internet of Things Innovation* (AIOTI) launched by the EU with the aim of strengthening links and building new relationships between the different IoT players (industries, SMEs, startups) [10]; the *Open Platform 3.0™* at The Open Group that focuses more on industrial applications and practices (https://www2.opengroup.org/ogsys/catalog/W145, last accessed October 2017); the *OneM2M* global standards initiative that involves eight standard bodies for Machine to Machine (M2M) communications [11]; the *IEEE Internet of Things (IoT)* initiative [12], or still the *International Technical Working Group on IoT-Enabled Smart City Framework* developed at NIST [13]. Although most of these initiatives promote various types of standards and technology enablers [14,15,16,17], they all share the same vision about relying as much as possible on open and interoperable standards in order to move towards open innovation ecosystems that have the capabilities to break down the vertical silos that shape today’s IoT.

Even if it is true that narrowing the range of IoT messaging standards, especially at the application layer, is a necessary step to enable the IoT to live up to its potential, there are other architectural and structural considerations to be taken into account. Amongst them, it is important to provide IoT stakeholders with easy-to-use tools, and most importantly incentive environments for publishing, searching for, and consuming various types of IoT data/services (A distinction is made between “IoT data” and “IoT services”. “IoT data” refers to data streams coming from sensors or other systems generating or holding data (database, file …), while “IoT services” refers to the call of a web service that takes, as inputs, one or more parameters and may imply a processing stage to return the expected result.) [18]. Such environments must also ease the control of personal data and business services, seeking to transform the current organisation-centric system to a human-centric one. Such a transformation was one of the reasons contributing to the development of the General Data Protection Regulation (GDPR) directive recently voted by the EC [19], in which rules are defined to both give citizens back control of their personal data and simplify the regulatory environment for businesses. All the above discussed aspects must be carefully tackled in any open innovation ecosystem, from its design to its development, use and management.

The recent IoT-EPI initiative (http://iot-epi.eu, last accessed October 2017), which is a joint project initiative between seven R&I (Research and Innovation) and two CSA (Coordination and Support Action) projects funded under the H2020 framework, is committed to make strides towards the creation of open and sustainable IoT ecosystems in Europe. This paper presents the architectural building blocks of the IoT ecosystem developed in one of these R&I projects. The paper is structured as follows: Section 2 discusses the implications and challenges of creating an open IoT ecosystem. Section 3 presents the technological building blocks underlying the presented ecosystem, with a specific focus on how to efficiently and productively access, find, share and compose new services/applications based on existing distributed/heterogeneous IoT endpoints. A real-life application in city of Lyon is then introduced in Section 4, in which the introduced ecosystem building blocks have been deployed and used by city system integrators. Discussion and conclusion are given in Section 5.

## 2. Towards Successful Open IoT Ecosystems—The European Ambition

**Figure 1:**
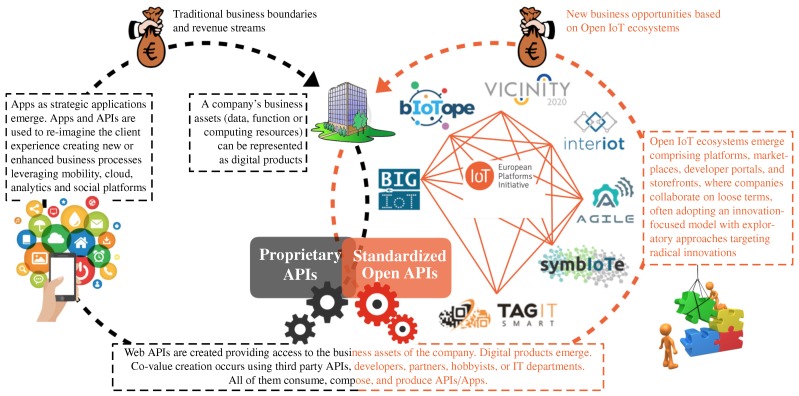
Towards open IoT ecosystems for joint service co-creation and radical innovation practices [5].

While in the US, IoT ecosystems are created around big, multinational players such as Google, Amazon, Facebook and Apple—*so-called GAFA* [20]—the EU’s strength is rather in smaller and agile companies [21]. In this respect, the EU launched various initiatives to foster the emergence of open innovation ecosystems enabling and incentivizing public and private institutions, as well as citizen communities, to join and contribute to the growth and sustainability of the established ecosystems. Among other initiatives, the AIOTI alliance (https://aioti.eu, last accessed October 2017), consisting of 13 Work Groups (focusing on security, standards, smart mobility, healthcare, etc.), was launched to make recommendations for future collaborative work in the context of the IoT Focus Area in the H2020 EU programme. As part of AIOTI, a sub-alliance named IoT-EPI (IoT-European Platforms Initiative), composed of 7 Research and Innovation (R&I) projects funded under the ICT30 cluster (2016–2019), has been initiated with the aim to turn existing vertically-oriented platforms and services into economically viable IoT ecosystems [5]. Figure 1 provides an at-a-glance overview of the desired impact from an “API economy” perspective [22,23], which, as stated by IBM [24]:

“The API economy has changed how we think about building applications (think apps) and how we deploy software (think cloud). The largest impact of this change for business is speed: Business processes and data are no longer locked inside applications. The result is the death of data and application silos.”

**Figure 2:**
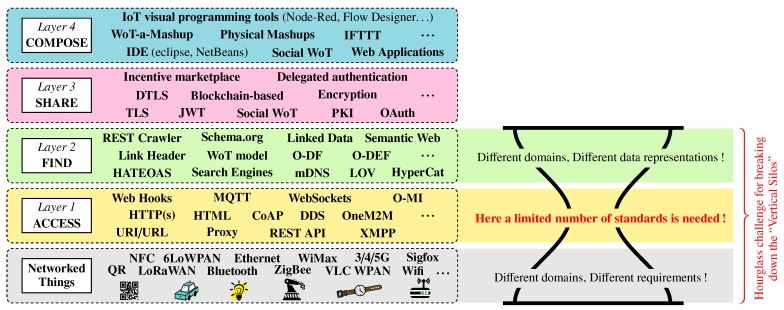
Key technological layers underlying open IoT ecosystems (adapted from [7]).

An important prerequisite for a successful open IoT ecosystem is to create a solid foundation, both technologically and economically viable, to ensure its take up by end-users. This includes establishing appropriate strategies to incentivize them in publishing and/or consuming IoT data/services, including the provision of secure and trustable services (e.g., with more open/intuitive solutions that help end-users to choose to start or stop sharing data) [19] to the provision of some kind of a digital marketplace through which end-users can trade personal data and/or services [25]. To accomplish this, it is important to define and implement the right building blocks to efficiently find, share and compose distributed and heterogeneous data sources in the IoT. This has been illustrated through the four-layer structure given in Figure 2, which has been adapted from [7]. Section 2.1, Section 2.2, Section 2.3, Section 2.4 and Section 2.5 discuss more thoroughly the ongoing research and development for each of these layers, along with challenges that remain to be solved for creating successful open IoT ecosystems.

### 2.1. Networked Things

Connecting every Thing to the Internet and giving them an IP address is only the first step towards the IoT, where the communication network can be a short-range radio (e.g., Bluetooth, ZigBee), a metropolitan area network (e.g., LoRa) or still a local Wi-Fi network in a building (e.g., Z-Wave, KNX). Building a single global IoT ecosystem to enable seamless communications between Things at this layer is virtually impossible, and does not even make sense as each protocol has been designed to meet different requirements (e.g., for firm, hard or soft real-time applications), often in different application domains.

### 2.2. ACCESS Layer

Unlike the Networked Thing layer, the application layer would require a single universal protocol to enable devices and applications to talk to each other regardless of how they are physically connected. This has been illustrated through the “hourglass” challenge in Figure 2. Nonetheless, at the time of writing this paper, many initiatives, pursued by distinct standardization fora, are currently underway, which inevitably slow down the convergence and adoption of a *de facto* application protocol for the IoT. Amongst these initiatives, there are two “schools of thought” if we may put it that way with:

1. the creation of new protocols that bypass traditional web protocols such as HTTP. Examples of well known protocols falling within this category are MQTT, CoAP or still the native XMPP protocol that directly rely on TCP or UDP;
2. the reuse and leveraging of widely popular Web protocols such as HTTP. Examples of such protocols are OneM2M, O-MI, Webhook that directly rely on or extend the HTTP protocol.

Even though a single universal application protocol is desirable for the IoT to become a reality, these two “schools of thought” once again address different requirements at the application layer. While the former specify protocols that are more suited to D2D (Device-to-Device), D2G (Device-to-Gateway) and D2C (Device-to-Cloud) communication models (considering the four IoT communication models defined in the RFC 7452) [26], the latter is more suited to the BDS (Backend Data Sharing) communication model. What cannot be disputed is the fact that the second school of thought gave rise to the so-called “Web of Things” (WoT), which uses and adapts Web protocols to give a digital presence to the Things on the World Wide Web [7]. We believe that these two categories of application protocols are likely the biggest hindrance to the mass adoption of IoT today, sometimes causing confusion in the academic and industrial sectors due to the wide range of standards today’s available. Given this situation, it remains difficult to speculate about which protocol(s)/standard(s) will remain or disappear in the future.

### 2.3. FIND Layer

Making Things accessible through a single universal application protocol does not ensure that applications can really “understand” what Things are about (i.e., the meaning and description of data and services they offer). This is where the second layer (FIND) plays a major role. This layer aims to describe Things in a way that enables automatic search, indexing, and integration of data/services exposed by these Things into applications. This issue is often described as a semantic interoperability challenge and mainly concerned with the payload of messages, which not only addresses the encoded data format, but further the semantic model that describes the meaning of the data. Furthermore, it is a prerequisite in order to understand the service description (i.e., to have a clear definition of what the service offers and how to request it).

Existing IoT platforms often rely on pre-defined data models to describe and annotate data that is generated by Things. However, such data models are technology-dependent and often inherent implementation-specific features (e.g., specific protocols, language support, structures, etc.), which affect the way and expressivity of the semantic definition [27]. In order to overcome these limitations and find a common approach that can be understood and extended by all stakeholders, Semantic Web [28] technologies are more and more often applied in IoT projects. The overall approach of applying ontologies to solve the semantic interoperability of the IoT is often described as the *Semantic Web of Things* (SWoT) [29]. The cornerstone of the semantic web is the Resource Description Format (RDF) which, when combined with other standards such as RDF Schema (RDFS) and Web Ontology Language (OWL), can represent knowledge about the physical world [30].

The fundamental idea to facilitate interoperability with semantics is to reuse and link to existing and commonly adapted ontologies of the corresponding domain, e.g., using the Semantic Sensor Network Ontology (SSN) [31] to describe sensor setups and readings. In practice, several repositories of such *Linked Vocabularies* have emerged in order to discover and promote the reuse of already defined terms and vocabularies, like the Linked Open Vocabularies (LOV) repository [32]. However, semantic-based approaches also face various challenges which are not yet solved. Firstly, creating and reusing semantic models for a specific domain is not an easy process, especially for non-experts of the semantic web [33]. Second, integration efforts of the Semantic Web principles to web services, like *Semantic Web Services* based on OWL-S [34], add even further complexity, which is an aspect that could contribute to the disruption and wide adoption of semantic-based approaches [35,36].

### 2.4. SHARE Layer

The role of this layer is twofold, specifying: (i) how the data generated by Things can be securely shared between data publishers and consumers; and (ii) how to incentivize stakeholders (developers, analysts, businesses and citizens) in sharing/consuming personal IoT data and/or business services.

Regarding the first role, one of the major concerns is the satisfaction of requirements in terms of network security (confidentiality, integrity, authenticity), identity management (authentication, authorization, accountability, revocation), data privacy (anonymity, pseudonimity, unlinkability), trust among users and Things, as well as resilience against attacks and failures [37,38,39]. This is of strategic importance within open innovation ecosystems since software producing organisations open their platforms to third parties, thus posing new challenges (e.g., malicious players may make use of vulnerabilities in the platforms, and vulnerabilities that were previously considered harmless are now open to the community) [38,40]. Privacy is also a major concern of any product and service consumer, where today’s models are mostly “organisation-centric” rather than “human-centric”, lacking of transparency and auditability [41,42]. The GDPR has the ambition to embrace such a paradigm shift by giving end-users back the control of their data [19], e.g., by informing end-users about what data has been collected, for what purposes and under which policies the data is going to be processed and retained, or still by enabling end-users to choose to start (or stop) sharing specific data/service items. Even though security and privacy mechanisms widely adopted by the Web community benefit IoT applications, such as TLS for making transactions secure, OAuth for authentication delegation, JWT for securely transmit information between parties, Public Key Infrastructure (PKI), or still blockchain-like technologies for maintaining a permanent and tamper-proof record of transactional data, there are still many challenges ahead that require further attention in IoT applications, as thoroughly discussed in [43,44,45,46].

Regarding the second responsibility (i.e., incentivizing IoT stakeholders to SHARE and consumes IoT data/services), there is a need to define and implement effective mechanisms for communities of developers and end-users to successfully engage with IoT ecosystems, thus featuring some challenges at the governance level. Indeed, while short-time collaboration is relatively easy to achieve in open ecosystems, it is much more challenging to define an efficient strategy to hit the intrinsic motivators of businesses/developers and end-users to achieve long-term engagement [47]. Open IoT ecosystems are expected and intended to support joint offerings, ad-hoc collaboration and co-creation between all categories of IoT stakeholders, thus following a value co-creation logic in which no fixed contracts between actors are established; collaboration is rather trust-based and agile [48,49,50,51]. A smart city is very concerned by this logic as it is a complex ecosystem comprising a wide range of stakeholders and service providers who must work together, including public administrations, network operators, energy providers, logistics and transportation centers, and so forth. To support trust-based and agile contracts, there is a need for the emergence of web environments that help publishers and consumers to securely expose, consume and even trade IoT data/services. Some platforms emerged in this regard, such as Dawex (https://www.dawex.com/en/), Placemeter (https://www.placemeter.com), Datacoup (http://datacoup.com) or still thingful (https://thingful.net), they cover different needs (Placemeter focusing on turning video into data, Thingful focusing on indexing IoT data/services without providing any reward in sight) and thus do not fulfill all prerequisites of open IoT ecosystems [5,52].

### 2.5. COMPOSE Layer

Once Things are published using a common/universal application protocol (ACCESS layer), where they can be found by humans and machines (FIND layer) and their resources be securely shared with third parties leading to potential rewards (SHARE layer), it becomes time to look at how to build meaningful cross-domain and/or cross-platform services. To put it another way, data and services coming from heterogeneous Things can now be easily, or at least with minimum interoperability issues, combined together and plugged to analytics software and mashup platforms in order to fulfil untapped applications across systems and application areas. Such a combination (COMPOSE layer) can be achieved using various IDE (Integrated Development Environment) solutions, ranging from web toolkits (e.g., JavaScript SDKs offering higher-level abstractions) to dashboards with programmable widgets, or still physical mashup tools such as ReactiveBlocks, Wyliodrin, Zenodys, AT&T Flow Designer, Node-RED. The latest tools, and particularly Node-RED, become increasingly used in both scientific and industrial communities, empowering ecosystem stakeholders to build applications without requiring advanced programming skills [7,53,54].

## 3. bIoTope Ecosystem: Architectural Building Blocks

**Figure 3:**
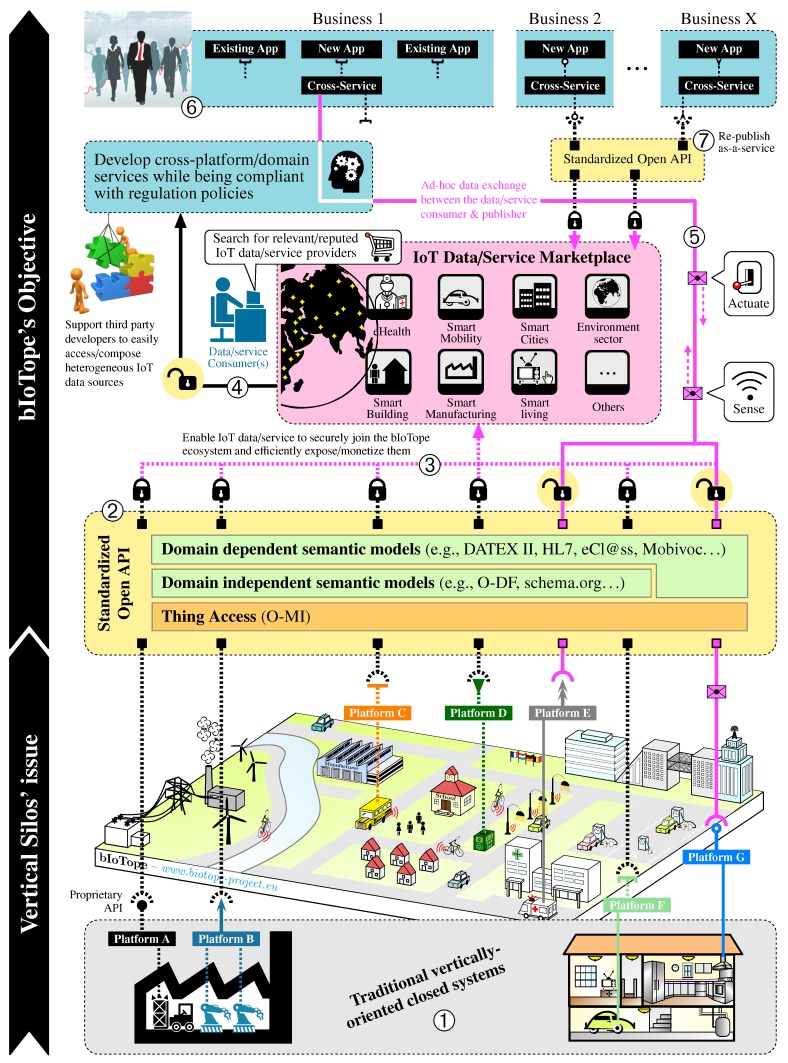
Overview of the building blocks of the bIoTope ecosystem based on four-layer structure given in Figure 2 (the color set introduced for each layer having been reused).

As part of the seven R&I projects composing IoT-EPI, the bIoTope project that stands for *“Building an IoT OPen innovation Ecosystem for connected smart objects”* (http://www.biotope-project.eu, last accessed October 2017) focuses on solving the “Hourglass challenge” previously introduced in Figure 2 (i.e., addressing the ACCESS and FIND layers). Also, a more in-depth overview of the bIoTope ecosystem is given in Figure 3, highlighting the key building blocks set up at each layer of the bIoTope ecosystem (the color set used for each layer in Figure 2 has been reused in Figure 3). Section 3.1 details the building blocks being specified and developed at the ACCESS/FIND layers, while Section 3.2 focuses on the ones at the SHARE/COMPOSE layer. Technologies used at the Networked Things layer is pilot-dependent, and thus will be discussed for the *Métropole de Lyon*’s pilot in Section 4.

### 3.1. Access & Find

Figure 2 shows—*through stage denoted by* ➀—that bIoTope leverages the available vertically-oriented platforms and cloud endpoints, thus following the logic discussed in Section 2.1 (i.e., not imposing any messaging protocol at the “Networked Things” layer).

From a data/service publication and consumption standpoint (see ➁ in Figure 2), bIoTope complies with the second school of thought by—*at the ACCESS layer*—leveraging HTTP to support more advanced operations such as subscription-like mechanisms. To this end, the bIoTope consortium selected the O-MI (Open-Messaging Interface) standard (https://www2.opengroup.org/ogsys/catalog/C14B, last accessed October 2017) published by The Open Group, which is protocol agnostic so they can be exchanged using HTTP, SOAP, SMTP, FTP or similar protocols. O-MI provides a standardized open API for implementing RESTful IoT information systems, supporting various types of subscription mechanisms (e.g., interval-based, event-based, with or without callback address, etc.) based on the Observer Design Pattern framework presented by [55] that makes it possible for Things to communicate with each other in a peer-to-peer fashion, thus differing from the Pub/Sub model that rely on brokers. In analogy to the Web that uses the HTTP protocol for transmitting HTML-coded information mainly intended for human users, O-MI is used for transmitting O-DF (Open-Data Format) (https://www2.opengroup.org/ogsys/catalog/C14A, last accessed October 2017) represented IoT information mainly for processing by information systems. Let us note that, in the same way as HTTP can be used for transporting payloads also in other formats than HTML (e.g., XML), O-MI can be used for transporting payloads also in other formats than O-DF. The O-DF standard fulfills the same role in the IoT as HTML does for the World Wide Web, meaning that O-DF is a generic content description model for Things in the IoT. O-DF can be seen as a first semantic enabler of the FIND layer, acting as a “domain independent semantic model” that can and should be extended with more specific semantic vocabularies (cf. Figure 2), both/either domain-independent vocabularies (e.g., iot.schema.org, SSN) and/or domain-dependent (e.g., DATEX II for the mobility sector, HL7 for the healthcare sector, WoT models, HyperCat, etc.).

Such an extension of O-DF has been discussed e.g., in [56]. Furthermore, a few examples of what O-MI/O-DF request/response messages look like can be found in [57,58,59], even though an example from *Métropole de Lyon*’s pilot will be presented in Section 4.

### 3.2. Share & Compose

**Figure 4:**
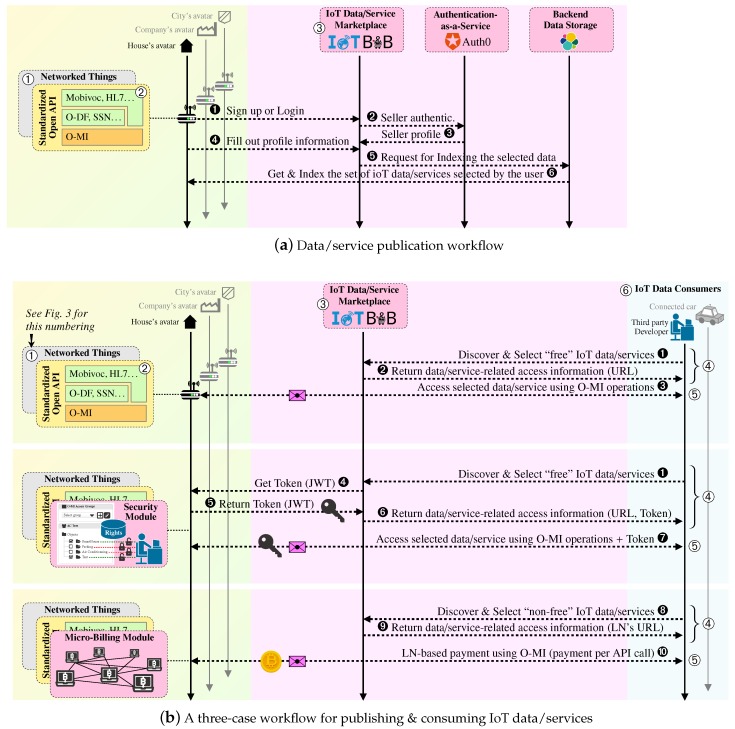
Data/Service consumers & publishers from the bIoTope ecosystem standpoint.

The SHARE and COMPOSE layers are in charge of incentivizing IoT stakeholders (e.g., developers, analysts, businesses, citizens) in sharing and consuming IoT data/services. Incentive schemes in bIoTope imply, among other prerequisites, to (i) support “human-centric” security models, seeking both to give citizens back control of their data and to simplify the regulatory environment for businesses, and (ii) enable data owners to easily participate in data trading [25]. To this end, an IoT data/service marketplace is currently being specified and developed in bIoTope, as illustrated in Figure 2. This marketplace, called IoTBnB (http://iotbnb.jeremy-robert.fr, last accessed October 2017) standing for IoT service puBlication and Billing, is intended to assist data owners (O-MI gateway owners to be more precise) in:

- choosing what data/service items they want to make available/visible to the user base engaged with the bIoTope ecosystem (cf. ➂ in Figure 2). Stages denoted by ➊ to ➏ in Figure 4a provides an overview of the IoTBnB back-end components, along with the different steps that gateway owners have to perform if they want to register to IoTBnB;
- specifying for which purpose the exposed data/service items can be used, for how long, and at what cost.

And furthermore assist data consumers (cf. ➃ in Figure 2) in:

- searching for valuable IoT data/service providers, enabling multimodal search like *(i) spatial/temporal search:* one may want to search for services within a geographical area; *(ii) keyword search:* one may want to search for services falling within a specific sector such as mobility, healthcare, environment, etc. *(iii) reputation search:* one may want to search for a service ensuring a certain quality level, which may depend on various dimensions: data provider reputation, data stream quality, etc. *(iv) contractual term or technology search:* one may want to search only for IoT data/service producers that make available data/service for free, or who are compliant with one or more crypto-currencies or still with specific license conditions;
- trading and negotiating for accessing one or more data/service items.

It should be stressed here that, at the marketplace level, only the “description” of what the data stream or web service is about, and how to call it, is collected and indexed by the O-MI search engine. Technically speaking, only the semantic models specified at the FIND layer of each IoT gateway is indexed in the service catalog, and not the underpinning data streams generated at the “Networked Things” layer. Indeed, once data consumers have found valuable data source descriptions via IoTBnB, agreed upon the terms and potentially paid for accessing the underlying resources, they can start communicating in a peer-to-peer manner with the corresponding IoT gateway(s), as illustrated through stage denoted by ➄ in Figure 2. So far, three distinct publication and consumption schemes are made possible in the bIoTope ecosystem, as summarized in Figure 4 (cf. Case 1–3).

**Figure 5:**
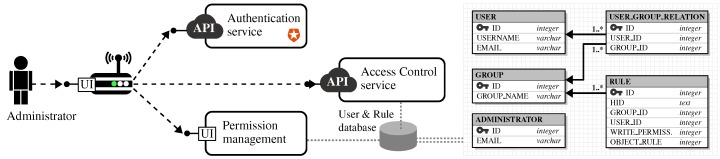
In-depth overview of the security module developed in bIoTope (cf. Case 2 in Figure 4b).

Case 1 corresponds to the basic publication/consumption scheme, where IoT data/services are published for free and without any access right. In this case, consumers search for relevant and valuable data/services on IoTBnB and then get the necessary information to access the underlying ressource (cf. arrows denoted by ➊ to ➌ of Case 1 in Figure 4b). Case 2 provides data/service publishers with the possibility to decide who can access what and how; this being specified at their gateway level (cf. “security module” in Figure 4b). When a consumer wants to directly access a secured data/service item whose URL is already known, she/he has to send a request to the gateway owner/administrator in order to be granted. In cases where the consumer discovers and selects the secured data/service item through IoTBnB, a token is generated by IoTBnB on behalf of the data/service owner (cf. arrows denoted by ➊, ➍–➏ of Case 2 in Figure 4b). Beforehand, the gateway owner/administrator must give full authority to the marketplace to manage the access to (or denial of) the data/service items when consumers have (or not) fulfilled the access/purchasing terms. This is done when the administrator registers her/his node to IoTBnB. Furthermore, to enable administrators to manage the who, what and how their data/services can be accessed by third parties, a complementary security module has been specified and developed in bIoTope, based upon five key requirements, which have been listed in Table 1. Figure 5 provides an overview of what the module consists of, namely: a ➀ *back-end database:* for storing user groups and related access rights; ➁ *external authentication service:* using the external web service Auth0; ➂ *User Interface:* enabling administrators to specify and manage access rights related to the registered users; ➃ *Access control service:* for checking what kind of permissions end-users have on the requested data/service. To avoid a scalability issue at the gateway level when managing a high number of users’ policies, part of this issue can be shifted to the (trusted) marketplace when registering a node, as the stateless JSON Web Tokens technology is used implying that the gateway only needs to generate and verify tokens when requested. This can be seen as one of the marketplace incentives to foster ecosystem participation. Case 3 aims to design a microbilling framework to help *(i) gateway administrators:* in evaluating the quality of their data/service items and thereby setting a reasonable purchase price; *(ii) data/service consumers:* in buying IoT data/services in ad-hoc, loosely coupled ways (cf. arrows denoted by ➒, ➓ of Case 3 in Figure 4b). Although Case 3 is not yet fully operational in bIoTope, first findings suggest that well-known decentralized currency technologies such as Bitcoin fail to meet key IoT requirements for efficient and scalable micropayments (e.g., ability to conduct fast transactions and scale massively in the number of users and payments). This is the reason why new decentralized ledger technologies are emerging, whether at the academic or industrial level, such as Duplex [60], Lightning Network (LN) [61], Inter-Ledger Protocol (ILP) [62], Greedy Heaviest-Observed SubTree (GHOST) [63], etc. As a starting point for experimentation, the bIoTope consortium decided to focus on the LN technology that allows for the creation of fast, scalable and ad hoc bitcoin-supported micropayment channels between users. Although some limitations still remain to be solved, LN is still under specification and associated reference implementations under development, therefore leaving room for innovation.

At the stage denoted by ➅ in Figure 3, third party consumers can rely on their own preferred IDE (Integrated Development Environment) for accessing and processing the selected data/services, meaning that bIoTope does not impose the use of a specific IDE tool. That being said, all software modules developed in bIoTope are made available in Node-RED.

## 4. bIoTope Ecosystem Serving as Interoperability Enabler Of “Smart *Métropole de Lyon*” Strategy

Predictions state that 66% of the world’s population will live into cities by 2050 [64], thus impacting how environment, housing, economy, transport and well-being of citizens is managed. The population of Lyon—*59 municipalities with an area of 538 km${}^{2}$*—is expected to increase up to 1.45 million inhabitants by 2030. This growth is accompanied by a rapid change in climatic conditions; the average temperature in Lyon having increased by $1.9{\;}^{\circ }$C between 1959 and 2017 [65]. In order to cope with this growth, *Métropole de Lyon* has drawn up a climate adaptation plan, along five strategic development and innovation axes: (1) *Preserving water resources:* by increasing from 78% to 85% of water supply network efficiency; (2) *Reducing urban heat-island (UHI) effects:* by increased the planting of new trees in the region (2000 to 3000 new trees per year); (3) *Better assisting the population:* by planning for heat waves with follow-up indicators; (4) *Adaptation of agricultural structures and practices:* accompanying measures to move towards a conservation agriculture; (5) *Improving local knowledge:* by better understanding the impact of climate change on local scales, understanding the local biodiversity/species and the needs of the territory and its inhabitants. Within the framework of bIoTope, a real-life pilot related to the second (UHI) axis is currently being developed, partly relying on the architectural building blocks presented in Section 3. Let us note that two other pilot cities (Brussels and Helsinki) are also part of the bIoTope project and ecosystem, thus making use of the different bIoTope building blocks previously discussed. Even if this paper focuses only on the most advanced pilot (i.e., Métropole de Lyon), comparisons between pilots will be carried out at the end of the project in order to evaluate and validate the extent to which one pilot (or part of the pilot) can be replicated in other cities.

Section 4.1 provides an overview of the work undertaken to renovate a whole street in Lyon (primary goal being to provide a wider pedestrian zone), from which *Métropole de Lyon* has benefited to conduct IoT research and experiments. In this section, first solutions deployed at the Networked Things layer are discussed and evaluated. Section 4.2 details the communication infrastructure (and associated performance) covering the ACCESS and FIND layers, while Section 4.3 focuses on the SHARE & COMPOSE layers.

### 4.1. Pilot context & Networked Things layer

**Figure 6:**
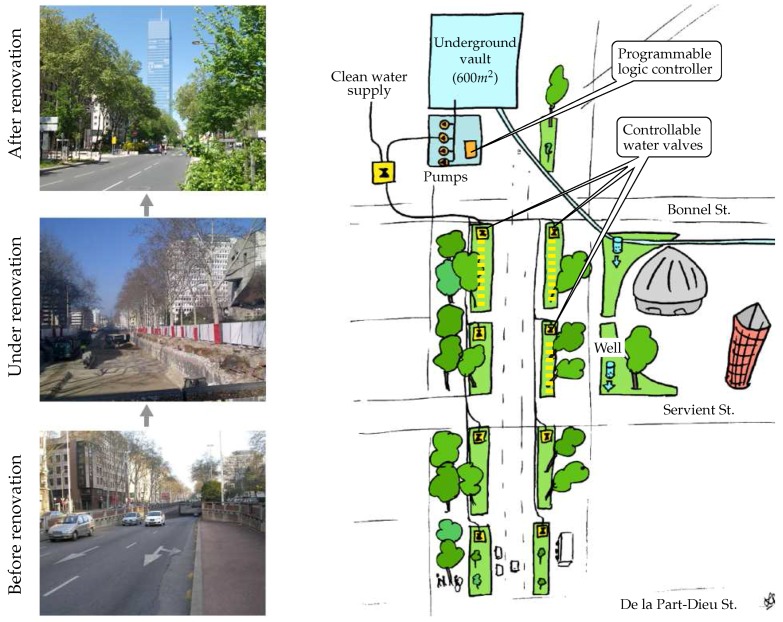
Overview of the Garibaldi St. transformation as part of a large-scale urban renewal project.

**Figure 7:**
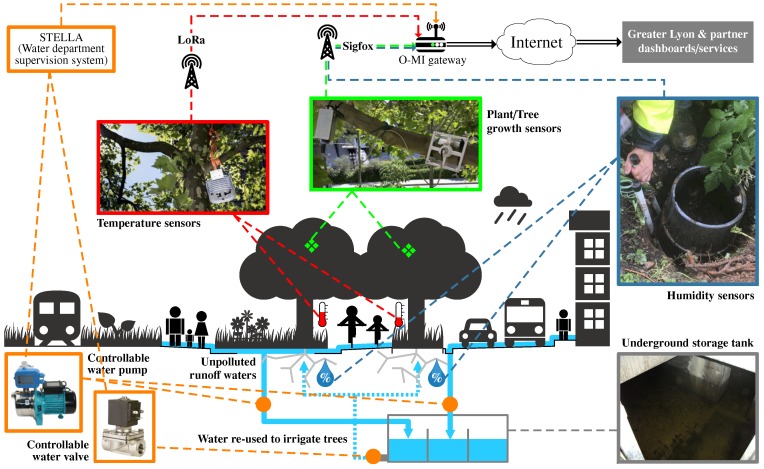
Communication infrastructure set up on Garibaldi St. to support the UHI project.

*Métropole de Lyon* has been carrying out UHI measurement campaigns using temporary or mobile sensors. However, partners of the territory, and in particular researchers focusing on the modelling of UHI phenomena and its impact on citizens, would like now to benefit from a true IoT network in order to carry out more rigorous studies. Within this context, the Garibaldi street, which is located in the heart of a business district in Lyon and is the subject of a large-scale urban renewal project, has undergone a fundamental transformation/renovation in 2014 in order to enable IoT experiments. The different steps of this transformation are shown in Figure 6 (left side) through three pictures showing the Garibaldi street before, under, and after renovation. An interesting feature of this street is that there was an underground vault of $1200\;m^{3}$, which has been repurposed to store rainwater which can be used to clean the streets, to refill street sweepers, or to water green areas. In this respect, and as highlighted in Figure 6 and Figure 7, the street-planted strips have water inlet connections to the pumps in this basin. During summer 2016, four green areas were equipped with various kinds of sensors, in particular, air temperature sensors (for UHI phenomenon analyses), soil moisture and tree activity/health sensors (for monitoring the watering needs of plants). Remote controllable pumps and valves (for irrigation control management) were also installed. These devices and associated communication protocols are summarized in Figure 7. Since this pilot is intended to be an experiment space, three distinct communication protocols are used, namely: *(i) LoRa network:* allows for the collection of temperature data (on an half-hourly basis); *(ii) Sigfox network:* allows for the collection of both soil moisture and tree activity data (on a half-hourly basis, too); *(iii) Proprietary STELLA network:* allows for the collection of water level data, as well as for the control of the water pumps and valves.

**Figure 8:**
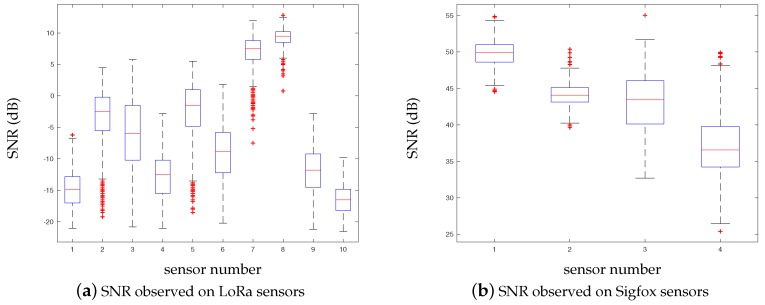
SNR observed on both LoRa and Sigfox sensor networks over a 2-month period.

**Figure 9:**
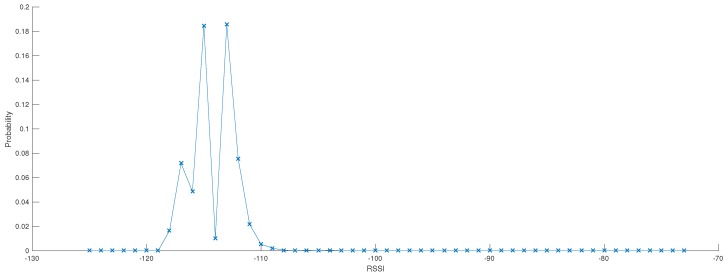
Probability-density function of RSSI (dBm) observed with LoRa sensors over a 2 month period.

Whenever a measure of temperature and tree growth is collected by the LoRa and Sigfox networks, the Signal-to-Noise Ratio (SNR) is measured and recorded. Even though the objective is not to compare LoRa and Sigfox technologies, it can be observed that the SNR values are better for Sigfox (cf. Figure 8b). On the other hand, LoRa technology does not necessarily imply to rely on a service provider/network operator; each person who owns a LoRa antenna/gateway can actually grant access to any third party. Such independency is an important aspect when dealing with/stepping into “open” IoT ecosystems. As a result, we have tested the LoRa network by installing our own LoRa gateways (Kerlink Wirnet Station 868 MHz ISM band, sensitivity up to −141 dBm) in the office of *Métropole de Lyon* with street temperature sensors within a 1 km radius (exact distances are given in Table 2). The objective of this experiment is to understand how the LoRa network/protocol behaves in urban environment settings. As shown in the Figure 8 but also in Figure 9, the small Received Signal Strength Indication—*RSSI*—(around −110/−120 dBm) and negative SNR values would suggest that the data was sent by devices that are located too far from the gateways. However, these results are mainly due to urban obstacles that impact on the signal intensity. Furthermore, we observed that the greater the radius distance between the antenna and sensor, the higher the daily packet loss.

In conclusion, it can be stated that LoRa antennas should be installed on high and secure points in order to decrease the number of packet loss, even though the criticality of this loss depends on the targeted applications.

### 4.2. Access & Find Layers

In this pilot, the temperature data collected by LoRa sensors are exposed through an O-MI/O-DF gateway, which runs on a physical machine having 2 processors (CPU E5-2640 @2.50GHz) and 48 GB of RAM. This machine is hosted in Lyon (France) behind a firewall. In this section, we propose to evaluate the behavior of the O-MI gateway—*mainly in terms of response time*—under heavy traffic load. To do so, a stress test is performed by using the open-source software Apache JMeter (Apache JMeter: http://jmeter.apache.org/, accessed on July 2017). This software allows to send concurrent requests to the O-MI gateway. Such requests were generated and sent from Luxembourg University’s network (from a APPLE Macbook Pro Retina—mid-2015—with an Intel Core i7 CPU @2.8GHz and 16 GB of 1600 MHz DDR3 RAM).

**Figure 10:**
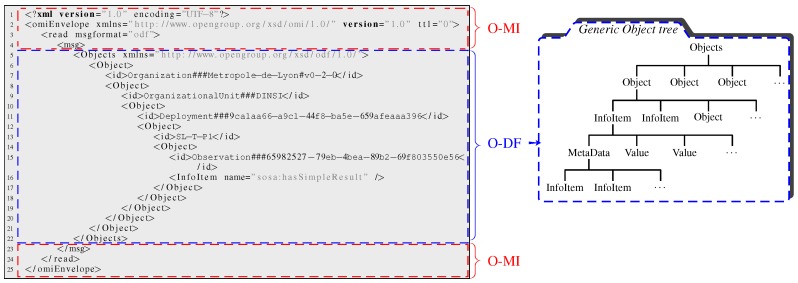
O-MI/O-DF: generic object tree and example of a O-MI/O-DF message relying on that tree.

**Figure 11:**
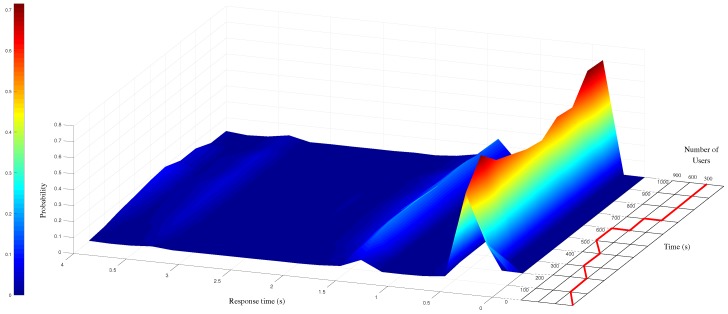
Probability-density function (95% of the response time values) considering the request given in Figure 10.

The test plan is designed as follows: simulated end-users send O-MI/O-DF requests to the gateway in order to receive temperature data measured by sensor number 1 (referred to as SL-T-P1 in the O-DF tree). The corresponding O-MI read request message (for each user) is displayed in Figure 10. Rows 1 to 4 (and 23 to 25) correspond to the message interface (i.e., O-MI-related parameters) whose requested operation is set to read. Rows 5 to 22 detail the message payload built on the generic O-DF information hierarchy (as summarized in the right-hand side of Figure 10). The instantiation made of this generic hierarchy by *Métropole de Lyon* highlights that Organization###Metropole-de-Lyon#v0-2-0’, OrganizationalUnit###DINSI and so on are defined as O-DF ’Object’ with specific IDs (cf. rows 7 and 9). The InfoItem (i.e., Object’s property) that needs to be read in that case is specified at row 16, namely the sensor 1’s value). As part of the FIND layer, it can be noted that the information hierarchy presented in this scenario is enriched with standardised vocabularies (linked open vocabularies to be accurate) such as “Sensor, Observation, Sample, and Actuator” (see acronym sosa at row 16), which contributes to ease the indexing and discovery process—*via IoTBnB*—of the associated information. Let us also note that any ontology can be used/integrated to the O-DF structure (e.g., SSN, SEAS, FOAF or GEO to name a few). The mapping between RDF, first-class citizen in semantics, and the O-DF structure, can be performed by applying the following simple rules: (i) RDF class members become O-DF Objects and (ii) RDF literals become O-DF InfoItems. Upon translating RDF into the O-DF structure, pretty much all the information is preserved except for the RDF properties linking RDF class members. As shown in Figure 11 (see curve in the right side), the number of concurrent users increases gradually, by groups of 300 users, up to 900 concurrent user requests on the same InfoItem. After 600 s, the number of users is decreased 300-by-300 until the end of the experiment (1000 s). This scenario was run only three times since the observed response times are similar in all the experiments. Figure 11 provides an aggregated view of the results as a probability-density function of 95% response time values. The remaining 5% corresponds to more important response times, which is likely due to either a bias introduced by the JVM (maximum memory defined for the application Jmeter exceeding) or a network infrastructure issue (e.g., due to a HTTP error code such as 502 Bad Gateway). As a result, this 5% are not considered in our study.

Based on the resulting response time, the following conclusions can be drawn:

- When the load is relatively low (i.e., between 1 and 300 concurrent users [0 s; 200 s]), the response time are mainly less than 0.5 s (i.e., more than 65% of the values);
- When the load increases (due to the increase of users [200 s; 600 s]), the probability of response time being equal to 0.5 s decreases accordingly (i.e., ≈50% of the values). In addition, the probability of having response times between 1 to 1.5 slightly increases. However, all the response times remains under 4 s;
- Finally, when the number of users is reduced back to 300, the server progressively adapts itself and the probability of short response time (≈0.5 s) increases to more than 65% of the values.

These response times are therefore suitable for slow dynamic systems that would be controlled over the Internet (i.e., with no hard real-time requirements). Let us note that the response times can be significantly higher when the size of the request increases (especially when a user wants to get several O-MI infoItems at once), therefore resulting in an increased number of TCP segments needed to generate and transport all data. To keep requests as small as possible, the developer first needs to search for—*through the marketplace*—InfoItems he/she would like to access to, therefore requesting a limited number of data on the corresponding IoT gateway(s).

### 4.3. Share & Compose Layers

**Figure 12:**
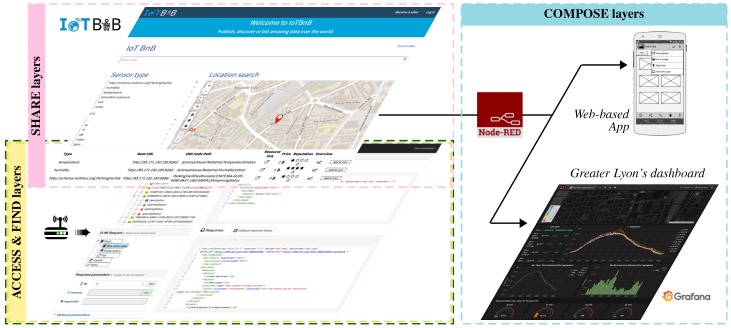
Screenshot overview of tools related to the ACCESS, FIND, SHARE & COMPOSE layers.

*Métropole de Lyon* is committed to provide public and private companies and organizations with the right infrastructure and conditions to easily and securely conduct disruptive projects. Given this commitment, the bIoTope ecosystem and associated service marketplace (IoTBnB) are used to allow combining heterogeneous data sources into different high-level services such as Web-based apps (e.g., for citizens) or dashboards (e.g., for city management department) in order monitor the behaviour of the Garibaldi street, as well as the “health” of the sensors (e.g., in terms of battery charge or signal quality). The reader can refer to the video tutorial available at https://www.youtube.com/watch?v=gYdFOVXd25o to get an overview about how IoT stakeholders can easily use bIoTope components/services made available through Node-RED (e.g., O-MI and O-DF nodes, IoTBnB node, etc.) in order to compose IoT workflows. All of this is summarized through Figure 12.

**Figure 13:**
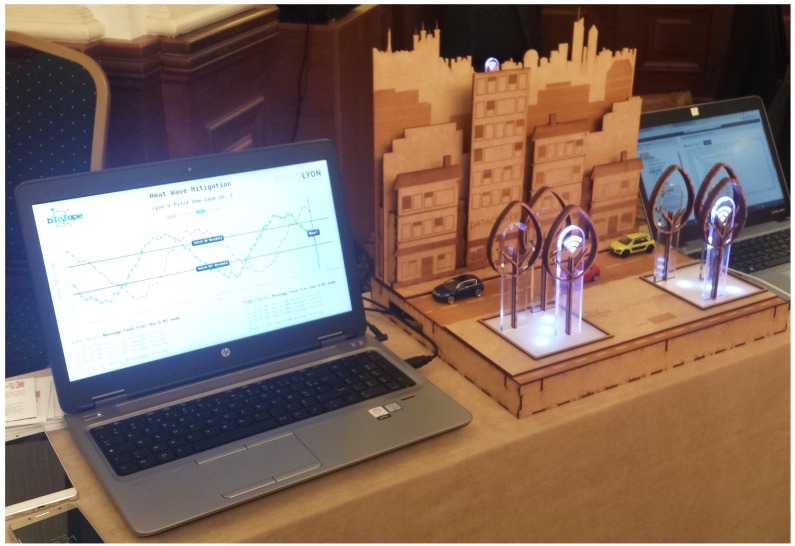
Physical mock-up designed by *Métropole de Lyon* as a proof-of-concept of the heat wave mitigation system built on the open IoT ecosystem (and related building blocks) developed in bIoTope.

The objective of *Métropole de Lyon* is twofold: *(i)* showing that standardized data can be easily discovered, accessed and composed; *(ii)* developing an irrigation system with the aim to mitigate heat island effects, thus increasing the citizens’ comfort. In this context, *Métropole de Lyon* has developed a physical mock-up of the Garibaldi street (see Figure 13) combining all data sources published by the O-MI gateway. It is composed of two distinct green areas contributing to the production of sensor data on air temperature, tree growth, etc. Data is collected at the “DATACENTER” building, standing for one of the buildings of *Métropole de Lyon*. For demonstration purposes, a simulated comfort indicator (based on simulated sensor data) is computed for each zone by the back-end system. Two simple control rules are applied, namely: *(i)* when the indicator exceeds the upper threshold, the system sends a command—*using an O-MI/O-DF write request*—to irrigate the area, which results in turning the (blue) LEDs located on the ground and *(ii)* when the indicator falls below the lower threshold, the system sends a command—*using an O-MI/O-DF write request*—to stop the irrigation. The evolution of the comfort indicator and the two thresholds are vizualized on the computer screen. In the Figure 13, it can be observed that the both comfort indicators (blue and red lines) exceed the upper threshold (even though the blue curve is between the lower and upper thresholds, the irrigation—cf. leds in the mock-up—is still active until the curve reaches the lower threshold), which is why both areas were “irrigated” (blue leds on the ground) when the picture was shot.

In this application, even though we showed that the irrigation system is feasible, it still remains to refine the control rule logic by relying on machine learning and domain-expert knowledge. For instance, it will be needed to analyse UHI patterns to verify whether additional irrigation can “boost” perspiration and keep trees in better health. Preliminary investigations show that, when trees are sufficiently hydrated, they evaporate water and, as a consequence, a better comfort level is perceived by the citizens. In the framework of Lyon’s pilot use case, such a control rule logic refinement (i.e., identifying the optimal indicators and valve opening/closing rules) will be achieved by one or more companies that will join the bIoTope consortium through open calls in early October 2017.

## 5. Conclusions

The IoT is revolutionising the digital landscape, making it possible for billions of devices to discover, communicate, and autonomously interact with each other for a wide range of smart applications. However, all these Things are today feeding vertically-oriented closed systems, architectures and application areas (commonly referred as “vertical silos”), which prevent the interaction between Things, thus entailing the development of competition in IoT markets and slowing down co-operative work between IoT stakeholders. This is all the more true in the context of Smart Cities, which are complex ecosystems comprising a wide range of service providers (network operators, energy providers, logistics and transportation centers, etc.) who must work together to meet societal and economic needs. Given this, and as part of an ongoing H2020 EU project (named bIoTope), the present paper discusses the EU’s vision and ambition to move towards successful and viable open IoT ecosystems.

To be technologically and economically viable, and to contribute to break down the vertical silos, open IoT ecosystems need to support key technological layers, described as the Access, Find, Share and Compose in this paper (cf. Figure 2). It is argued that, to create a true and federated IoT, the most critical layer is the Access one for which a common agreement about a single universal protocol needs to be reached. However, there is still a fierce competition between standardization fora, making it difficult to to speculate about what standard(s) will remain or disappear in the future. In the framework of the bIoTope project, two open communication standards named O-MI and O-DF are considered at the Access and Find layers respectively. The smart city pilot, jointly developed with *Métropole de Lyon*, shows that these two standards enable to meet the prerequisites of an open IoT ecosystem. Pilots in Brussels and Helsinki cities are currently under development, which will be used as comparison basis for evaluating and validating the extent to which one pilot (or part of the pilot) can be replicated in other cities.

Future research work is concerned with the integration of contextual data in the ecosystem (e.g., benefiting from semantic web technologies at the Find layer) in order to design and support more advanced and “smart” services. In addition of this technological proof-of-concept, much remains to help cities and business to step into open IoT ecosystems from a business perspective. To this end, bIoTope proposes an IoT data/service marketplace (called IoTBnB), which opens up new forms of Business-to-Business and Customer-to-Business interactions. Indeed, IoTBnB seeks to provide IoT data/service producers and consumers with an easy-to-use and secure environment for publishing and/or consuming (even trading) IoT data and services. Such marketplace environments can greatly contribute to transform the current organisation-centric system to a human-centric one, which is aligned with the EU General Data Protection Regulation (GDPR) directive 95/46/CE.

## Figures and Tables

**Table 1:** Key requirements covered by the O-MI security and Micro-billing modules (Case 2 in Figure 4).

Requirements	Description
Access control of resources	Only users having access rights can perform O-MI/O-DF request actions over the data/service tree.
Group-based rules	All end-users must belong to one or more groups, for which access rules must be specified.
Operation-based permission	A permission (based on the possible O-MI verbs: e.g., read-only or read-write) can be specified for each data/service item and depending on the user group.
Recursive permission	Permission is inherited from the parent’s Object as well as overridden for particular children.
Management interface	The gateway administrator must be able to manage access rights through a centralized user interface.

**Table 2:** Features of LoRa sensors and daily packet loss over a 2-month period.

Sensor n^o^		1	2	3	4	5	6	7	8	9	10
Radial Dist.		699 m	506 m	440 m	510 m	353 m	376 m	254 m	158 m	381 m	739 m
	min	0 (0%)	0 (0%)	0 (0%)	0 (0%)	0 (0%)	0 (0%)	0 (0%)	0 (0%)	0 (0%)	0 (0%)
Packet loss	avg	11 (23%)	1 (2%)	3 (6%)	7 (15%)	1 (2%)	4 (8%)	1 (2%)	1 (2%)	5 (11%)	17 (36%)
	max	33 (69%)	3 (6%)	15 (31%)	26 (54%)	4 (8%)	18 (38%)	3 (6%)	3 (6%)	20 (42%)	44 (92%)
